# Angiostrongyliasis, Mainland China

**DOI:** 10.3201/eid1110.041338

**Published:** 2005-10

**Authors:** Xiao-Guang Chen, Hua Li, Zhao-Rong Lun

**Affiliations:** * Southern Medical University, Guangzhou, People's Republic of China; † Zhongshan (Sun Yat-Sen) University, Guangzhou, People's Republic of China

**Keywords:** angiostrongyliasis, epidemiology, foodborne disease, emerging infections, letter

**To the Editor:** The first case of angiostrongyliasis caused by *Angiostrongylus cantonensis* in mainland China was reported in 1984; only 3 cases were reported between then and 1996 (*1*). Recently, however, cases of angiostrongyliasis have increased rapidly because of its natural focus and a change in human dietary patterns. For example, snails have become a popular food in many regions of this country. Nearly 100 cases of angiostrongyliasis have been reported in mainland China, including 2 outbreaks (*2**,**3*).

From 1994 to 2003, 84 cases of angiostrongyliasis were documented in mainland China. Of all the cases, 29 were reported individually, and 55 were reported from the 2 outbreaks that occurred in Zhejiang and Fujian. Sixty-three of the 84 patients had eaten raw or undercooked snails, 5 had eaten raw crabs, 1 swallowed tadpoles, and several pediatric patients had close contact with snails. Some researchers believe that the larvae of *A. cantonensis* can be released from mollusks into slime fluid and contaminate produce and other objects as they crawl. However, Liang et al. (*4*) reported finding no larvae in body fluid washed from 23 *Achatina fulica* that were infected with *A. cantonensis*. Therefore, whether slime fluid plays a role in human infection remains unclear.

The clinical symptoms of the patients from the 2 outbreaks of angiostrongyliasis that occurred in China are shown in the Table. Eosinophilia is a typical characteristic of eosinophilic meningitis or meningoencephalitis caused by *A. cantonensis.* In our study, eosinophilia was detected in 79 of 84 patients. Therefore, clinical patients with eosinophilic meningitis or meningoencephalitis with eosinophilia should be presumptively considered to be infected with *A. cantonensis* and parasitologic or serologic tests should be performed. The parasitologic detection rate of *A. cantonensis* infection in humans is low; because this parasite is found in the human central nervous system (CNS) and the tiny larvae often stick to meninges or nerve root, a false-negative result is often shown when cerebrospinal fluid is examined. In the 84 cases reported in this article, worms were isolated from only 8 patients (9.5%), whereas 64 cases were diagnosed as angiostrongyliasis by immunologic methods. The most common immunologic methods used to diagnosis angiostrongyliasis in this country are indirect fluorescent antibody test, immunoenzymatic staining technique, and enzyme-linked immunosorbent assay. All antigens used in these methods were prepared from whole adult worms. Reports indicated that serologic cross-reaction occurred between trichinosis and angiostrongyliasis when whole-worm lysate was used as the antigen (*5*). In addition, whole-worm antigens cannot discriminate between new and previous infection, or monitor the efficacy of the treatment. Therefore, finding potential diagnostic antigens will be essential to solving this problem and applying recombinant antigens may achieve this goal. A cDNA library from the larvae of *A. cantonensis* was constructed and screened with acute infection sera, and a diagnostic antigen that can detect early infection of *A. cantonensis* (3 weeks) was identified (*6*).

**Table:** Clinical characteristics of patients with angiostrongyliasis in 2 outbreaks in China*

Characteristics	Outbreak 1 (%)	Outbreak 2 (%)
No. of patients	47	8
No. of male patients (%)	24 (51)	8 (100)
Age, y		
Median	32	12
Range	6–58	11–13
Incubation (day)		
Median	10.35	6
Range	1–27	5–7
Symptoms†, no. (%)		
Headache	44 (93.6)	8 (100)
Nuchal rigidity or neck pain	0	6 (75.0)
Fatigue	7 (14.9)	7 (87.5)
Vomiting	9 (19.1)	8 (100)
Paresthesias	30 (63.8)	3 (37.5)
Muscle pain	43 (91.5)	8 (100)
Fever	27 (57.4)	3 (37.5)
Cough	4 (8.5)	0
Somnolence	4 (8.5)	7 (87.5)
Skin eruption	10 (21.3)	0
Skin itch	13 (27.7)	0
Laboratory detection No. positive/no. examined (%)		
Eosiniphils in cerebrospinal fluid	23/25 (92)	8/8 (100)
Eosinophils in blood	23/25 (92)	8/8 (100)
Serologic diagnosis	21/25 (84)	NT
Pathogenic test	ND	1/8‡ (12.5)

*NT, not tested; ND, not detected. †No patients had visual disturbance or photophobia, hyperesthesias, muscle weakness, or diarrhea. ‡Two larvae were found in cerebrospinal fluid.

Some researchers considered that anthelminthics, such as albendazole, ivermectin, mebendazole, and pyrantel, did not affect *A. cantonensis* but noted that the death of worms in the CNS might exacerbate neurologic symptoms (*7*). However, many studies in mainland China showed that anthelminthics can relieve symptoms and reduce the duration of disease. For example, Wang et al. (*8*) reported that albendazole could relieve the symptoms of angiostrongyliasis and suggested that it can be used to treat the disease. Lin et al. (*3*) also reported that in 8 patients who were treated with 20 mg/kg albendazole for 9 days, the symptoms and signs of acute angiostrongyliasis were rapidly relieved in 3–6 days. All of these patients had recovered by 10 days after treatment, and no side effects were observed.

Angiostrongyliasis is an emerging foodborne public health problem in mainland China. However, most clinicians are not familiar with this disease and little is known about the prevalence of *A. cantonensis* in China. Thus, more studies should be conducted on the biology, epidemiology, and clinical characteristics of angiostrongyliasis, and more effective diagnostic methods and treatments for *A. cantonensis* should be developed.
